# Use of Dexmedetomidine in Early Prone Positioning Combined With High-Flow Nasal Cannula and Non-Invasive Positive Pressure Ventilation in a COVID-19 Positive Patient

**DOI:** 10.7759/cureus.10430

**Published:** 2020-09-13

**Authors:** Elis M Cruz Salcedo, Lyd-Marie Rodriguez, Jay Patel, Andrew R Seevaratnam

**Affiliations:** 1 Internal Medicine, HCA/University of Central Florida Consortium, Ocala Regional Medical Center, Ocala, USA; 2 Pulmonary and Critical Care, HCA/University of Central Florida Consortium, Ocala Regional Medical Center, Ocala, USA

**Keywords:** covid 19, prone positioning, self prone, ards (acute respiratory distress syndrome), covid-19 respiratory failure, high flow nasal cannula, non-invasive positive pressure ventilation, dexmedetomidine

## Abstract

As the COVID-19 pandemic continues to manifest in our society, we still lack evidence-based treatment guidelines. Current treatment for COVID-19 pneumonia has been modeled from currently established guidelines such as that of acute respiratory distress syndrome (ARDS). COVID-19 pneumonia, also known as SARS-CoV-2, is characterized by severe hypoxia and near-normal respiratory system compliance with a time-related presentation. Dexmedetomidine is a centrally acting alpha-2 receptor agonist that promotes sedative and anxiolytic effects without the risk of respiratory depression and can provide cooperative or semi-rousable sedation. Patients who are developing ARDS secondary to COVID-19 pneumonia have been treated with self-proning intervals in combination with supplementation of oxygenation via high-flow nasal cannula (HFNC) or non-invasive positive pressure ventilation (NIPPV); however, a few patients have poor tolerance to the devices, leading to poor compliance and eventual worsening respiratory symptoms leading to intubation. In the current case report, we detail how a patient was able to successfully be self-proned with proper tolerance to HFNC and NIPPV while using dexmedetomidine, leading to discharge without the need for further oxygen supplementation at home.

## Introduction

As the COVID-19 pandemic continues to manifest among our society, we still lack evidence-based treatment guidelines. To this day, a wide array of treatment modalities have been used, recorded, and disseminated amongst health care providers detailing their management and results. Current treatment for COVID-19 pneumonia has been modeled from currently established guidelines such as that of acute respiratory distress syndrome (ARDS). Communication between physicians who have been directly involved in the care of COVID-19 positive patients has been key to establish and tailor current management recommendations by focusing on different theories of pathophysiology affecting the lungs. COVID-19 pneumonia, also known as SARS-CoV-2, is characterized by severe hypoxia and near-normal respiratory system compliance, with a time-related presentation. Two phenotypes have been identified and been described as Type L and Type H [1]. Both presentations differ in pathophysiology, particularly as Type L can evolve into type H, ultimately meeting the Berlin criteria of ARDS [1]. Different treatments have been proposed per pathophysiology type. For the initial stages of Type L, an increase in the fraction of inspired oxygen (FiO_2_) administered with non-invasive ventilation methods, such as high-flow nasal cannula (HFNC) has improved the clinical outcome of some patients [1]. It has also been noted that for these patients, prone positioning decreases intubation rates and improves outcomes for those developing ARDS [2-3]. This combination of early prone positioning combined with non-invasive options of ventilation may have an association with a reduced intubation rate and prevention of transition to phenotype H [1]. Dexmedetomidine is a centrally acting sedative and anxiolytic, which may promote relief of anxiety from dyspnea and promote adherence to respiratory support from HFNC and NIPPV [4]. Our case details a patient with COVID-19 pneumonia who was successfully managed with awake self-proning while using dexmedetomidine in combination with HFNC and noninvasive positive pressure ventilation (NIPPV) during the beginning stages of the disease in order to promote increased adherence to proning cycles and oxygen support equipment, to prevent endotracheal intubation.

## Case presentation

**Figure 1:**
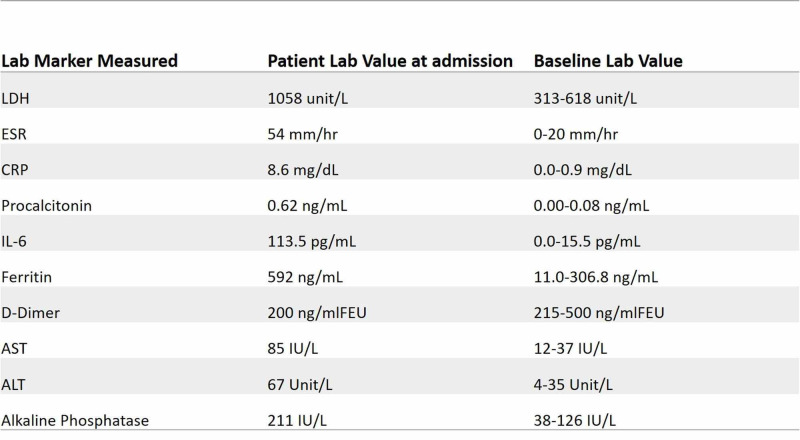
Significant laboratory markers and their reference value for a patient with COVID-19 pneumonia

**Figure 2:**
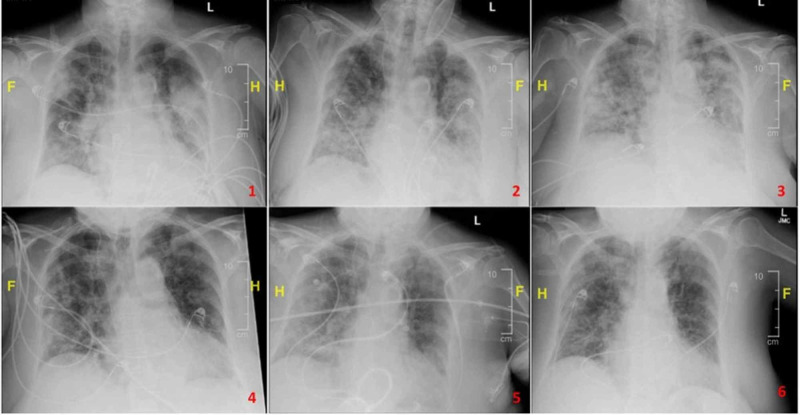
Serial portable chest X-rays demonstrating COVID-19 pneumonia lung-related changes 1: day 1 (admission); 2: day 3; 3: day 5; 4: day 7; 5: day 9; 6: day 11

A 73-year-old, non-smoker Caucasian female with a history of asthma, hypothyroidism, hypertension, and right breast cancer currently on remission, presented with complaints of worsening fatigue, myalgia, productive cough of clear sputum, and 101℉ temperature that began one week prior to presentation. There was no travel history but the patient had a positive history of contact with a close relative with similar symptoms. On presentation, she was afebrile, with a heart rate of 99 beats per minute, respiratory rate of 22 breaths per minute, and oxygen saturation at room air of 92%. The examination was remarkable for diminished breath sounds bilaterally. Laboratory studies showed a leukocyte count of 3,900 cells/µL with a normal differential and a coagulation panel within normal limits. Further laboratory marker results for lactate dehydrogenase (LDH), erythrocyte sedimentation rate (ESR), C-reactive protein (CRP), procalcitonin, interleukin 6 (IL-6), ferritin, and d-dimer are detailed in Figure 1. Chest X-ray showed bilateral lung infiltrates and a venous Doppler ultrasound for deep vein thrombosis was negative. Severe sepsis criteria were met on admission, with evidence of end-organ damage and elevated troponin I of 0.14 ng/mL and a creatinine level at 1.0 mg/dL from a baseline of 0.5 mg/dL. The patient was started on azithromycin 500 mg intravenous (IV) once daily and ceftriaxone 1,000 mg IV once daily in addition to fluid resuscitation pending COVID-19 PCR results. Final blood cultures and the influenza test were negative, however, the COVID-19 PCR test was positive. Initial hypoxemia resolved with oxygen supplementation with a 2 L oxygen nasal cannula but progressively worsened requiring supplementation with a 50% FiO2. Arterial blood gas at that time showed acute hypoxic respiratory failure, prompting an escalation of treatment to HFNC and transferring the patient into the medical ICU. Hydroxychloroquine 400 mg once daily, methylprednisolone 40 mg every eight hours, and enoxaparin 1 mg/kg subcutaneous twice daily were added to the medical regimen. A repeat chest X-ray showed progressive worsening of bilateral infiltrates. Considering worsening hypoxemia, intermittent self-proning was instituted with alternating HFNC and NIPPV. Dexmedetomidine was initiated to assist with self-proning tolerance. Proning sessions lasted an average of four hours alternating between supine and prone positions. Ceftriaxone was discontinued and cefepime 1,000 mg every eight hours was initiated. The patient also received one unit of convalescent plasma. Experimental therapies with tocilizumab and remdesivir were attempted but were unable to be provided. She completed a five-day course of azithromycin and a 10-day course of hydroxychloroquine. Slow interval improvement on images (Figure 2) and in the clinical presentation was noted, resulting in improvement of oxygenation and eventual discharge home without requirement for oxygen supplementation following a 17-day hospital stay.

## Discussion

To the best of our knowledge, this is one of the few cases reported in the United States of treatment with awake self-proning combined with HFNC and NIPPV, with the use of dexmedetomidine. Prior studies have demonstrated how HFNC is associated with improved survival rate among patients with acute hypoxemic respiratory failure when compared to standard oxygen or noninvasive ventilation; and it is associated with an increased degree of comfort, a reduction in the severity of dyspnea, and a decreased respiratory rate [5]. Prior studies have shown how prone positioning has a mortality reduction and improved oxygenation when applied early and for prolonged time periods in patients with severe ARDS [6]. Unlike proning intubated patients with ARDS, self-proning is less labor-intensive and poses a lower occupational hazard risk to nursing staff since patients are able to conduct this themselves. There is evidence that the aforementioned strategy resulted in the discharge of a COVID-19 patient without requiring intubation in Ontario, Canada [7]. Additionally, a case series report of patients from a New York City hospital hypothesized an association between proning patients with COVID-19 and improvement in oxygen saturation [2]. The addition of dexmedetomidine sedation was essential to our management with self-proning, as it allowed adherence to treatment. Dexmedetomidine is a centrally acting, presynaptic selective alpha-2 adrenoreceptor agonist that inhibits the release of norepinephrine from synaptic vesicles, which promotes analgesic, anxiolytic and sedative properties [4]. By including the use of dexmedetomidine for patients using HFNC or NIPPV, it helped promote compliance and comfort during supine-prone rotation cycles and allowed improved tolerance to an otherwise previously oxygen support-naïve patient. Additionally, isolation measures for COVID-19 positive patients place them at a higher risk tier for anxiety, both from dyspnea-associated anxiety and isolation anxiety from strict distancing requirements in order to prevent further dissemination of the disease. This case further contributes to the current literature, aiming to increase recognition and to incite further investigations on potential modalities of treatment of COVID-19 pneumonia, with a special emphasis on reducing intubation rates, reducing mortality, patient comfort, and preservation of resources.

## Conclusions

Dexmedetomidine is a centrally acting alpha-2 receptor agonist that promoted sedative and anxiolytic effects without depressing the respiratory system. In patients who require increments of oxygen supplementation without the need for endotracheal intubation and/or those who present with the inability to tolerate HFNC or NIPPV, experience anxiety associated with dyspnea, or are unable to be compliant with self-proning, dexmedetomidine may be used effectively to assist with compliance and tolerance. Patients who are able to tolerate self-proning treatment in combination with HFNC and NIPPV may experience improvement in oxygenation, leading to clinical improvement and discharge home without oxygen requirements.
